# MicroRNAs Regulated by Pregnancy Target Antiviral and Cancer Immunity Overlapping with the HIV Interactome

**DOI:** 10.3390/v18070753

**Published:** 2026-07-07

**Authors:** Paula F. T. Cezar-de-Mello, Jonathan M. Dreyfuss, Pai-Lien Chen, Hidemi Yamamoto, Xiaoming Gao, Hui Pan, Charles Morrison, Gustavo F. Doncel, Robert L. Barbieri, Raina N. Fichorova

**Affiliations:** 1 Brigham and Women’s Hospital, Harvard Medical School, Boston, MA 02115, USA; pmello@bwh.harvard.edu (P.F.T.C.-d.-M.); hyamamoto@bwh.harvard.edu (H.Y.); rbarbieri@bwh.harvard.edu (R.L.B.); 2 Joslin Diabetes Center, Harvard Medical School, Boston, MA 02215, USA; 3 FHI 360, Durham, NC 07701, USA; pchen@fhi360.org (P.-L.C.); sophiexmgao@gmail.com (X.G.); charlessmorrison2@gmail.com (C.M.); 4 CONRAD, Eastern Virginia Medical School, Norfolk, VA 23507, USA; doncelgf@odu.edu

**Keywords:** pregnancy, breastfeeding, non-coding RNA, miRNA, viral infections, cancer cell cycle

## Abstract

Innate immunity predictors of HIV-1 risk and pathogenesis vary with reproductive hormones, pregnancy, and lactation, yet the underlying mechanisms remain unclear. We hypothesized that pregnancy-associated physiological adaptations alter systemic microRNA (miRNA) expression, thereby regulating immunity, pathogenesis and susceptibility to infection. We analyzed 174 serum samples from 88 participants in a longitudinal cohort from Uganda and Zimbabwe across pre-pregnancy (PP), pregnancy (P), and postpartum breastfeeding (BF). Cell-free peripheral blood miRNAs (n = 2083) were profiled using HTG EdgeSeq. Pregnancy-specific miRNAs were identified by intersecting differentially expressed (DE) miRNAs from P vs. PP and P vs. BF comparisons. miRNA targets and pathways were analyzed using miRWalk, Cytoscape/ClueGO, and cytoHubba. Pregnancy was associated with DE miRNAs (29 upregulated and 131 downregulated) targeting 2733 validated genes. Enriched pathways (FDR < 0.05) included adaptive immune response, Hippo Signaling, Cellular Senescence, HSV-1 infection, and two cancer-related pathways. Pregnancy-enriched targets within each pathway overlapped with the HIV–host interactome by 37–88%. Network analysis identified 47 hub genes interacting with 18 HIV-1 proteins, with Tat and gp120 being most connected viral and HLA-A being the most connected host protein. These findings indicate that pregnancy-driven systemic miRNAs target the HIV–host interactome and specifically identify pregnancy-enriched central hub genes involved in cell cycle control, viral immune evasion and replication to be further investigated for their predictive value in HIV acquisition and pathogenesis in longitudinal cohorts and experimental settings.

## 1. Introduction

Compelling evidence shows that the likelihood of HIV acquisition is elevated during pregnancy and the early postpartum period [1]. Reducing HIV acquisition requires comprehensive interventions, including early identification of risk factors and a deeper understanding of the immune mechanisms involved. HIV pathogenesis, disease progression and transmission are also affected by pregnancy, although negative outcomes are significantly reduced in combination with highly active antiretroviral therapies [2,3]. During pregnancy, the maternal immune system adapts to tolerate the fetus while maintaining protection against infections, with trimester-specific changes in complement activation, complement regulatory proteins, and levels of B cells, T regulatory cells, and neutrophils as well as shifts in CD4+ HIV target cell frequencies and activation [4,5,6,7]. However, the molecular mechanisms driving these changes and underlying HIV acquisition and disease progression during pregnancy remain poorly understood.

Non-coding RNAs emerge as important regulators of maternal health [8]. Among these, miRNAs—particularly those originating from the placenta—have been associated with pregnancy complications, inflammation, and fetal tolerance [9]. miRNAs are ~22-nucleotide long non-coding RNAs that control gene expression by binding to the 3′ untranslated regions of target mRNAs, leading to either inhibition of translation or degradation of the mRNA [10]. miRNAs are present not only within cells but also as cell-free molecules in body fluids such as blood, serum, and urine, where they are carried in extracellular vesicles or bound to proteins like Argonaute 2 that protect and transport them [11]. The intercellular exchange of miRNAs represents a mechanism of systemic gene regulation between distant cells, extending their function beyond intracellular control. Also, their presence as cell-free miRNAs in blood makes them potential biomarkers of physiological and pathological states [12]. There is evidence that circulating cell-free miRNAs can enter various immune cell lineages, including HIV host cells, leading to measurable changes in gene expression [13], and can also be carried to recipient cells in the peripheral tissues and mucosal surfaces through extracellular vesicle-mediated transport [14], thus presenting various plausible mechanisms for affecting susceptibility to disease.

Studies on pregnancy-associated miRNAs in relation to HIV susceptibility remain limited. miRNAs have been linked to several viral infections, including HIV [15], and more recently, circulating miRNAs have also been associated with the regulation of key drug-metabolizing enzymes and transporters [16]. miRNAs, as key regulators of immune and viral pathways, represent a potential mechanistic link between pregnancy-related immune adaptations and heightened HIV susceptibility and progression. This study employed a global miRNA transcriptomic approach combined with comprehensive functional bioinformatics analysis to investigate the role of miRNAs differentially expressed (DE) during pregnancy in HIV acquisition risk within a high-risk cohort from sub-Saharan Africa [17]. We specifically analyzed the overlap of the targets of miRNAs associated with pregnancy and those within the HIV interactome, defined as the set of host genes with experimentally validated roles in HIV pathogenesis.

## 2. Materials and Methods

### 2.1. Study Population

Participants of this nested study were part of a larger multicentric cohort on hormonal contraception and HIV acquisition (HC-HIV study) conducted in Uganda and Zimbabwe [18]. A total of 88 HIV-negative women who later became pregnant were enrolled in the HC-HIV and were included in this nested study, providing 174 serum samples collected in 3-month intervals. Women were tested at each study visit for *Candida albicans* and a panel of sexually transmitted infections (STIs) consisting of human immunodeficiency virus (HIV), herpes simplex virus (HSV), *Chlamydia trachomatis*, *Neisseria gonorrhoeae* and *Trichomonas vaginalis* [19,20]. The presence of bacterial vaginosis (BV) was also assessed at each visit by experienced physicians, using the Nugent score [19]. Table 1 summarizes population characteristics and clinical variables.

### 2.2. Biospecimen Collection and Processing

Peripheral blood was collected by venipuncture and cell-free serum was separated by a standard technique and centrifugation before aliquoting and freezing. The frozen specimens were transported to Fichorova’s Laboratory of Genital Tract Biology at Brigham and Women’s Hospital, which operates under accreditation by the College of American Pathologists following the standardized procedure for processing and storage at −80 °C until miRNA analysis.

### 2.3. Whole Human miRNA Transcriptome Profiling

The miRNA whole transcriptome (miRWT) from serum samples of women in pre-pregnancy, pregnancy, and breastfeeding stages was analyzed using the high-fidelity HTG EdgeSeq platform (HTG Molecular Diagnostics Inc., Tucson, AZ, USA). This platform uses a quantitative nuclease protection assay combined with PCR to attach adapters and barcodes, enabling an RNA extraction-free procedure as previously described [21]. Following sample cleanup, a library pool was generated and amplified for next-generation sequencing (NGS) using the Illumina NextSeq instrument and the NextSeq 500/550 High Output Kit v2.5 (75 cycles). Samples were processed across three batches of miRNA transcriptome assay, which included 2083 mature miRNAs, 13 housekeeping genes, a positive control (Table S1), and 5 negative control genes (from *Arabidopsis thaliana aintegumenta*: ANT1, ANT2, ANT3, ANT4, and ANT5) [21]. The HTG miRWT covers most genes of the placenta-expressed chromosome 19 miRNA clusters C19MC (33/63) and miR-371-3 (3/6) and the chromosome 14 miRNA cluster C14MC (91/92).

### 2.4. Differential miRNA Expression and Statistical Analysis

Low-expressing miRNAs were filtered out by retaining those with counts per million (CPM) > 4 in at least 10 samples, rendering 2079 miRNAs. Voom transformation [22] was applied to convert counts to log-CPM, accounting for mean-variance relationships and assigning precision weights based on read depth. The log-CPM values were normalized using quantile normalization [23]. Principal component analysis (PCA) was performed using the R package stats (v4.3.1) to assess the similarity and dissimilarity among samples based on their miRNA expression profiles. miRNAs DE between groups (pre-pregnancy, pregnancy, and breastfeeding) were identified using mixed-effect linear models using the *dream* function (R package variancePartition v.1.30.2) [24], and tested for fixed (group, country, plate, Nugent score, candidiasis and STIs) and random-effect (participant ID) variables. Missing values for candidiasis, Nugent score, and *T. vaginalis* were imputed due to inconsistencies between visits. If a value was missing at a later visit but available at the first visit, it was imputed using the previous visit’s data. If the first visit had missing data, the value was imputed using data from the subsequent visit. We calculated moderated t-statistics for differential expression using the *eBayes* function, which applies empirical Bayes moderation to shrink standard errors toward a global mean. Volcano plots label top miRNAs by the smallest *p*-values or the largest log-FC. Heatmaps plot clipped z-scores of adjusted log-CPM for top miRNAs selected by *p*-values across comparisons.

### 2.5. Identification of Genes Targeted by the DE miRNA

DE miRNA (False-Discovery Rate, FDR < 0.1) in the comparisons between Pregnant vs. Pre-pregnant and Pregnant vs. Breastfeeding groups were overlapped to identify miRNAs that were up- and downregulated during pregnancy. The miRNAs (miRBase V22) were queried in the miRWalk database (release_2022_01) [25] to identify empirically validated target genes. The identified targets were annotated [26] and then utilized for Modular Enrichment Analysis (MEA). All comparative set analyses were conducted using Venn diagrams generated with InteractiVenn [27], while the UpSet plot [28] was created using the UpSetR package (V1.4.0) [29].

### 2.6. Construction of an HIV-Interactome Dataset

We created an HIV-interactome gene dataset by combining human genes identified in a previous study [30] with those from the manually curated “HIV-1 Human Interaction Database” [31]. The database was maintained by the National Center for Biotechnology Information (NCBI) until June 2024 and downloaded by us on 2 October 2023. It compiles reports on HIV–human protein–protein interactions (“protein interactions”) and HIV replication and infectivity (“replication interactions”). The Luo et al. report comprises 237 genes, while the “HIV-1 Human Interaction Database” contains 4577 genes, with an overlap of 211 genes. Thus, our HIV-interactome dataset includes 4603 genes for analysis (Table S2). The HIV-interactome genes were annotated using the SYNGO ID conversion tool [26].

### 2.7. Modular Enrichment Analysis

All the validated predicted targets of DE miRNA in pregnancy were subjected to Modular Enrichment Analysis (MEA) by applying ClueGo v2.5.9 [32] and CluePedia v1.5.9 (3), which were implemented as Cytoscape (v3.10.3) applications. The MEA identifies biologically meaningful patterns by analyzing groups (modules) of related genes or proteins. It employs *Kappa* statistics and *p*-value calculations to assess enrichment, accounting for the redundant and networked nature of biological annotations [33]. The upregulated (Cluster 1) and downregulated (Cluster 2) gene sets were loaded into ClueGo. Non-redundant biological information was obtained from Gene Ontology of Immune System (GOIS) and Kyoto Encyclopedia of Genes and Genomes (KEGG) by merging redundant groups with ≥50% gene overlap and preserving the more representative terms per their association strength (*Kappa* score ≥ 0.6). The statistical significance of enriched terms was estimated by enrichment/depletion two-sided hypergeometric test and corrected by the Bonferroni step-down test (*p*-value cutoff ≤ 0.05). HIV-interactome genes contributing to enrichment were mapped by overlapping with the HIV-interactome dataset constructed as described above. Then, they were annotated using the AutoAnnotate app (v1.5.2) [34], and hub genes were identified through topological analysis of the protein–protein interaction (PPI) networks using the cytoHubba (v0.1) [35] as described [21]. PPI and hub gene-targeting miRNAs networks were visualized with the Organic Layout algorithm (yFiles Layout Algorithms, v1.1.4), where nodes represent biological entities (e.g., genes and proteins) and edges denote the interactions or relationships between them. Maximum Clique Centrality (MCC) scores from high to low were assigned to identify the most crucial hub proteins. Predicted interactions between HIV-interactome, hub genes and their associated enriched pathways were uploaded to Cytoscape, and the network was visualized using Degree Sorted Circle Layout, followed by the generation of a hub elements subnetwork using cytoHubba. The subnetwork was visualized using Organic Layout.

## 3. Results

### 3.1. Serum miRNA Expression Changes Across Stages of Female Reproductive Status in Longitudinal Samples

**Figure 1:**
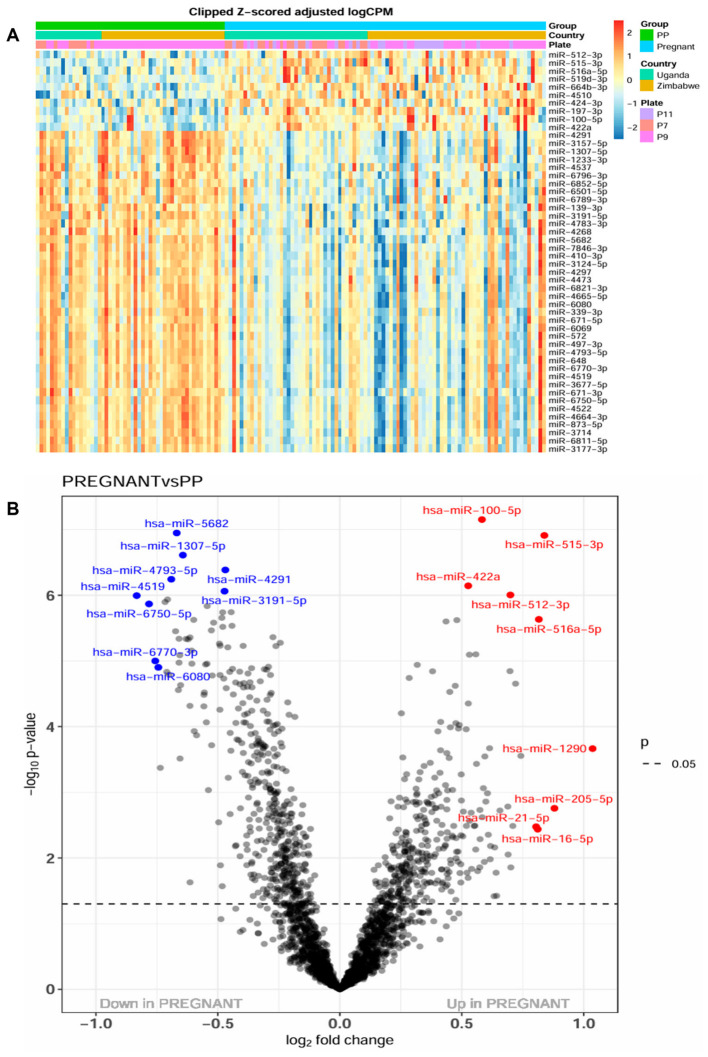
Serum miRNA expression by female reproductive status comparing pregnancy (P) versus pre-pregnancy (PP). (**A**) A heatmap displays the top 50 DE miRNAs (rows) across individual samples (column) for the comparison P vs. pre-pregnancy PP with clustering by country and batch effect (plate). Expression levels are represented by color intensity: red for upregulation and blue for downregulation. (**B**) A volcano plot shows differentially expressed (DE) miRNAs for P vs. PP. The most significantly downregulated (blue) and upregulated (red) miRNAs based on the smallest *p*-values or largest log fold changes (log FC) are annotated.

**Figure 2:**
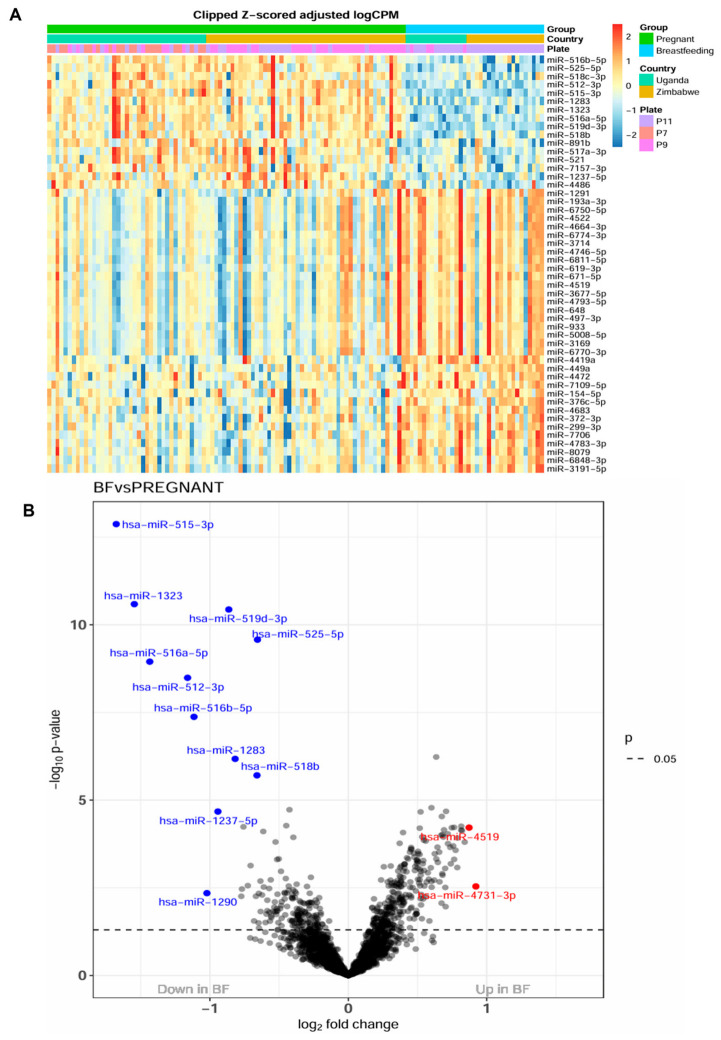
Serum miRNA expression by female reproductive status comparing breastfeeding (BF) versus pregnancy (P). (**A**) A heatmap displays the top 50 DE miRNAs (rows) across individual samples (column) for the comparison of BF vs. pregnancy P with clustering by country and batch effect (Plate). Expression levels are represented by color intensity: red for upregulation and blue for downregulation. (**B**) A volcano plot shows differentially expressed (DE) miRNAs for BF vs. P. The most significantly downregulated (blue) and upregulated (red) miRNAs based on smallest *p*-values or largest log fold changes (log FC) are annotated.

The PCA plots comparing sample variance showed that the samples from pre-pregnant (Supplemental Figure S1A) and breastfeeding (Supplemental Figure S1B) women displayed modest separation from those of pregnant women, while the total variance explained by PC1 (20%) and PC2 (12%) was low, suggesting lack of systematic bias between the comparison groups and warranting searching for a defined set of differentially expressed genes as biomarkers of reproductive condition. Pregnancy vs. Pre-pregnant comparison revealed 684 DE miRNAs and Pregnancy vs. Breastfeeding revealed 240 DE miRNAs with FDR < 0.1) (Table S3), corresponding to 32.8% and 11.5% of the miRWT whole transcriptome, respectively. Heatmaps illustrate the top 50 DE miRNAs across samples for the Pregnancy vs. Pre-pregnancy (Figure 1A) and Breastfeeding vs. Pregnant (Figure 2A) comparisons. Volcano plots annotate the miRNAs with the lowest FDR and the largest fold change in both comparisons (Figure 1B and Figure 2B).

Pregnancy-driven serum miRNAs identified by the overlap between the Pregnant vs. Pre-pregnant and Pregnant vs. Breastfeeding comparisons included 160 DE miRNAs (FDR < 0.1), of which 29 were upregulated and 131 downregulated (Table S4). Among those DE miRNAs, 19 belonged to the placenta-expressed clusters C19MC (upregulated hsa-miR-517a-3p, -516a-5p, -516b-5p, -525-5p, -519d-3p, -518c-3p, -512-3p, -522-3p, -1283, -521, -515-3p and -1321), C14MC (downregulated hsa-miR-410-3p, -376c-5p, -377-3p, -299-3p, -299-5p, and -1247-5p) and hsa-miR-371-3 cluster (downregulated hsa-miR-372-3p).

### 3.2. Pregnancy-Driven Serum miRNAs Target Genes Shared with the HIV Interactome

**Figure 3:**
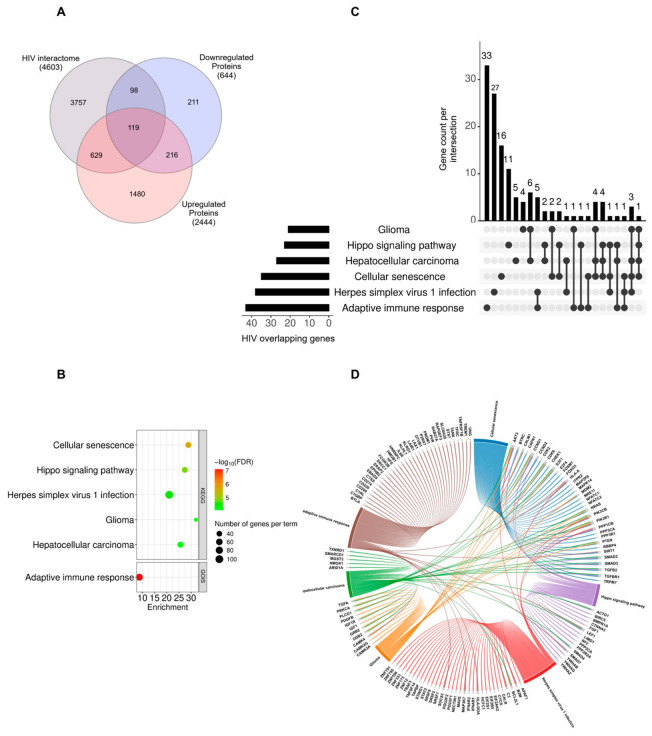
Modular enrichment analysis (MEA) of validated targets of serum miRNAs altered during pregnancy and enriched in the overlap with the HIV interactome. (**A**) A Venn diagram shows the overlap (intersections) and unique elements (differences) between the HIV interactome and the validated targets of pregnancy-driven serum miRNAs. (**B**) A bubble plot presents MEA of all validated targets (2444 upregulated and 644 downregulated) of pregnancy-driven serum miRNAs. The Y-axis annotates the names of enriched pathways (KEGG, upper plot) and terms (GOIS, lower plot) and the X-axis shows enrichment as % of genes per pathway or term. Bubble color represents the significance level of each pathway/term (-log10 FDR), while size indicates the total number of genes enriched in each pathway/term. (**C**) An UpSet plot and (**D**) a cord plot highlight the HIV interactome gene overlap contributing to the enrichment of the biological pathways shown in (**B**). The Y-axis of upper graph on the UpSet plot shows the count of HIV-overlapping genes that are either unique to or shared among specific pathway combinations, as indicated by the connected dots below the X-axis. Horizontal bars on the lower left of the Upset plot represent the total number of HIV-overlapping genes associated with each individual enriched pathway. The chord plot illustrates all genes within enriched biological terms that overlap with the HIV interactome and their distribution across pathways. Ribbons and colors in the cord plot distinguish the 6 enriched biological groups/pathways. The percentages below the labels indicate the proportion of genes in each term that are shared with the HIV interactome.

Pregnancy-driven DE miRNAs were predicted to downregulate 644 genes and upregulate 2444 genes, based on miRNA-mRNA regulatory relationships (Table S5). Among them, 98 (15.2%) of downregulated genes and 629 (25.7%) of upregulated genes overlapped with the HIV interactome dataset (Figure 3A), while 119 proteins fell into the intersection of the three groups and 216 were predicted to be bidirectionally expressed (both up- and downregulated) (Figure 3A, Table S6).

### 3.3. The Genes Targeted by Pregnancy-Driven Serum miRNAs Are Linked to Pathways of Adaptive Immune Response, Hippo Signaling, Cancer, Cellular Senescence and Viral Infection Shared with the HIV Interactome

All confirmed targets of pregnancy-modulated serum miRNAs were employed to identify associated biological pathways. Targets were found to be functionally associated with “adaptive immune response” (GO:0002250), “Cellular Senescence” (KEGG:04218), “Hippo Signaling Pathway” (KEGG:04390), “Herpes Simplex Virus 1 (HSV1) Infection” (KEGG:05168), “Glioma” (KEGG:05214) and “Hepatocellular Carcinoma” (HCC) (KEGG:05225) with an FDR < 0.05 (Figure 3B). The GOIS term “adaptive immune response” was the most significant (corrected *p* = 0.000015) while “Cellular Senescence” showed the greatest enrichment score (28.85% of genes per pathway) (Figure 3B, Table S7). Over 71% of the genes associated with those six biological functions are predicted to be upregulated as a result of downregulated pregnancy-driven DE miRNAs (Table S7). Among the genes contributing to enriched biological pathways, 132 were found to overlap with the HIV interactome (Figure 3C, Table S8), highlighting shared molecular mechanisms. Of these, 36 genes are shared across the six key pathways identified (Figure 3C,D, Table S8).

The differential miRNA-targeted HIV interactome genes were unevenly distributed between the six pregnancy-enriched pathways (Figure 3C and Table S8). Viral infection and immune response contained a higher number of HIV-interactome genes not functionally shared with the other terms (27 in “HSV-1 infection” pathway and 33 in “adaptive immune response”). Functional connections between the DE miRNA target genes within the enriched biological pathways (Figure 3D) showed that cancer-related pathways and “Cellular Senescence” functionally share the largest number of HIV-interactome genes. The percentage of genes shared with the HIV interactome within each pathway ranged from 37% to 88% (Tables S8 and S9).

### 3.4. Protein–Protein Interaction (PPI) Network Mapping of the Pregnancy-Enriched HIV Interactome Reveals Hub Gene Interactions While miRNA–hub Gene Networks Highlight Pregnancy-Driven miRNA Control of These Hub Genes

**Figure 4:**
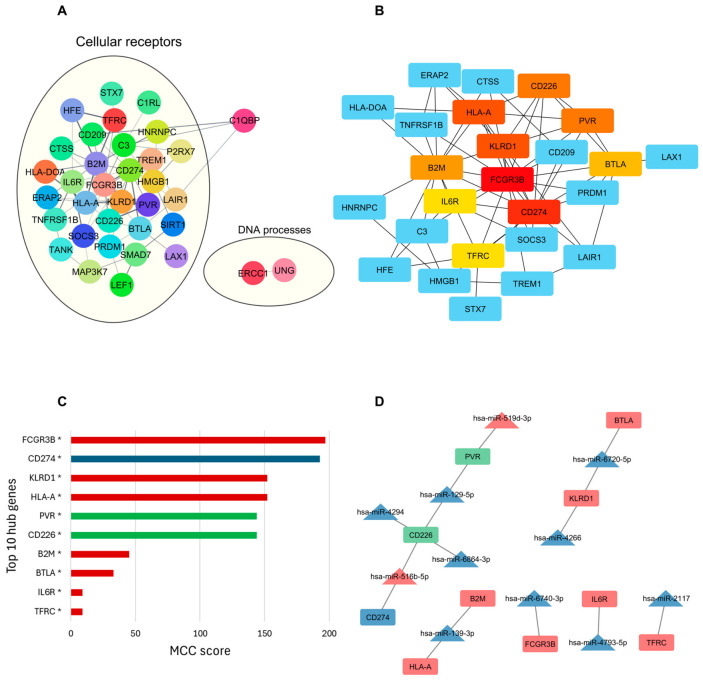
Protein–protein interaction (PPI) and hub gene networks of HIV-interactome genes within the adaptive immune response GO term, including predicted miRNA–target interactions. (**A**) Protein–protein interaction (PPI) networks display HIV-interactome genes contributing to the adaptive immune response term enrichment. Functional annotations (cellular responses and DNA processes) highlight commonalities in protein roles. (**B**) PPI network of the top 10 hub genes within the term, identified using cytoHubba based on Maximum Clique Centrality (MCC) analysis. Node colors indicate MCC scores, from high (red) to low (yellow). (**C**) Bar plots of the top 10 hub genes by MCC score. Asterisks indicate genes annotated under cellular receptor-related functions, as shown in Figure 4A. Bar colors represent the predicted regulation of each gene in pregnancy based on differential miRNA expression: blue for downregulated, red for upregulated, and green for mixed regulation (both up- and downregulated). (**D**) Networks of miRNAs and their hub gene targets show 21 nodes (triangles) and 15 edges (lines). Triangles represent miRNAs and rectangles represent hub genes. Node colors reflect inferred regulation in pregnancy: blue (downregulated), red (upregulated), and green (mixed).

**Figure 5:**
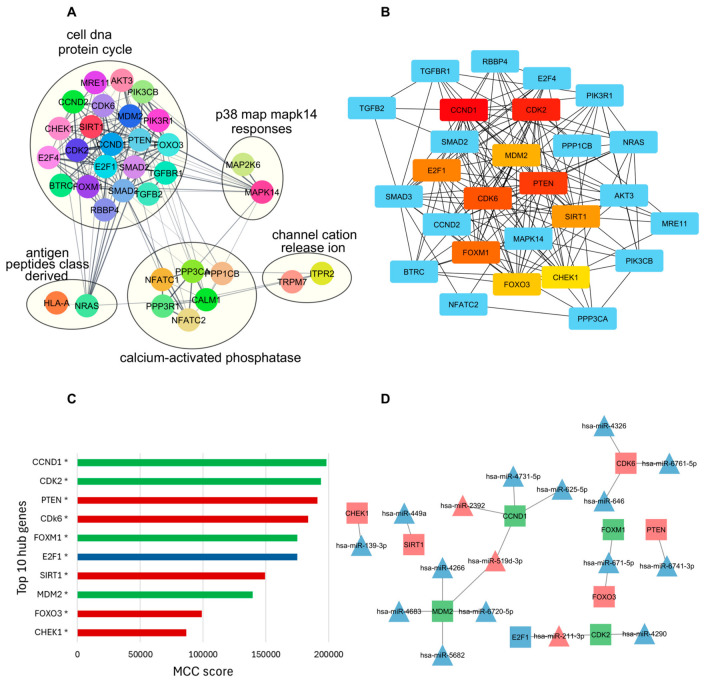
PPI and hub gene networks of HIV-interactome genes within the Cellular Senescence KEGG pathway, including predicted miRNA–target interactions. (**A**) Protein–protein interaction (PPI) networks display HIV-interactome genes contributing to the cellular senescence pathway enrichment. Functional annotations highlight commonalities in protein roles. (**B**) PPI network of the top 10 hub genes within the term, identified using cytoHubba based on Maximal Clique Centrality (MCC) analysis. Node colors indicate MCC scores, from high (red) to low (yellow). (**C**) Bar plots of the top 10 hub genes by MCC score. Asterisks indicate genes annotated under a cell cycle, as shown in Figure 5A. Bar colors represent the predicted regulation of each gene in pregnancy based on differential miRNA expression: blue for downregulated, red for upregulated, and green for mixed regulation (both up- and downregulated). (**D**) miRNA–hub gene target networks show 27 nodes (triangles and rectangles) and 20 edges (connecting lines). Triangles represent miRNAs and rectangles represent hub genes. Node colors reflect inferred regulation in pregnancy: blue (downregulated), red (upregulated), and green (mixed).

**Figure 6:**
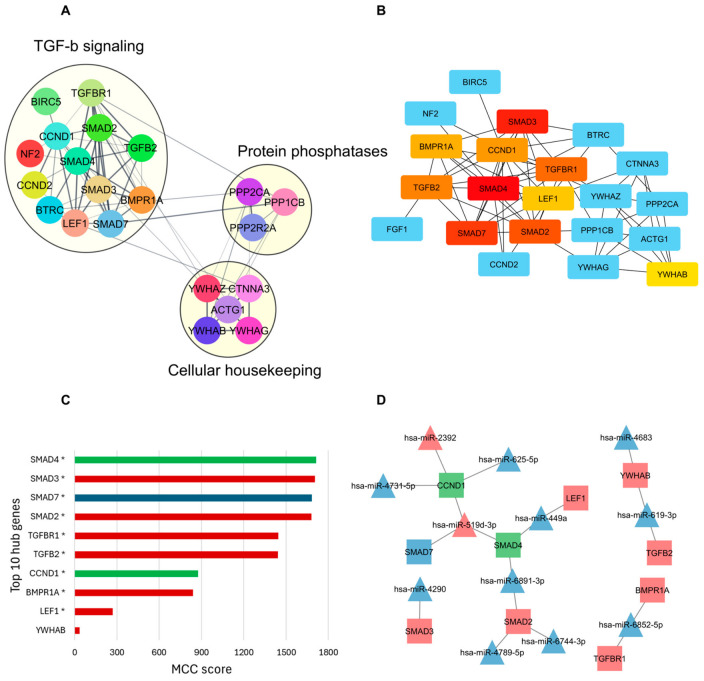
PPI and hub gene networks of HIV-interactome genes within the Hippo Signaling KEGG pathway, including predicted miRNA–target interactions. (**A**) Protein–protein interaction (PPI) networks display HIV-interactome genes contributing to the Hippo Signaling pathway enrichment. Functional annotations highlight commonalities in protein roles. (**B**) PPI network of the top 10 hub genes within the term, identified using cytoHubba based on Maximal Clique Centrality (MCC) analysis. Node colors indicate MCC scores, from high (red) to low (yellow). (**C**) Bar plots of the top 10 hub genes by MCC score. Asterisks indicate genes annotated under TGFβ signaling, as shown in Figure 6A. Bar colors represent the predicted regulation of each gene in pregnancy based on differential miRNA expression: blue for downregulated, red for upregulated, and green for mixed regulation (both up- and downregulated). (**D**) miRNA–hub gene target networks show 22 nodes (triangles and quadrangles) and 18 edges (connecting lines). Triangles represent miRNAs and rectangles represent hub genes. Node colors reflect inferred regulation in pregnancy: blue (downregulated), red (upregulated), and green (mixed).

**Figure 7:**
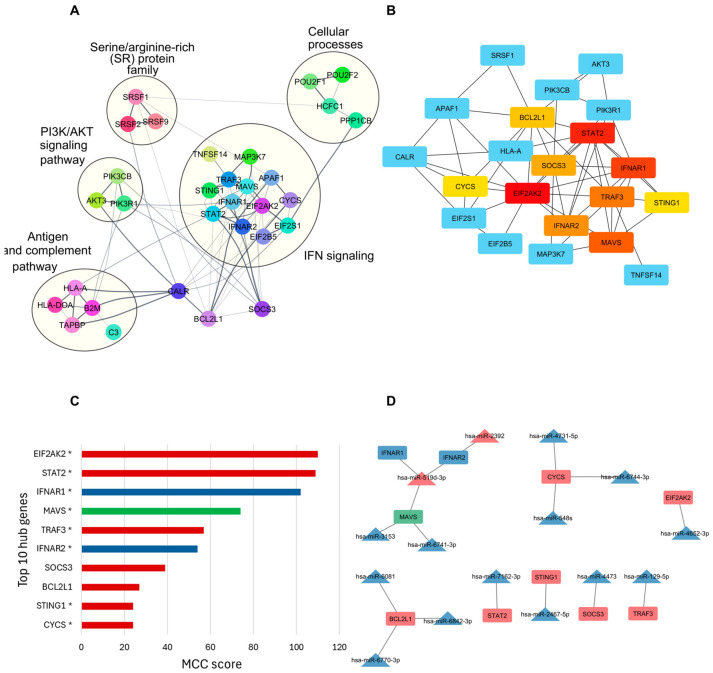
PPI and hub gene networks of HIV-interactome genes within the herpes simplex virus 1 Infection KEGG pathway, including predicted miRNA–target interactions. (**A**) Protein–protein interaction (PPI) networks display HIV-interactome genes contributing to the herpes simples virus 1 (HSV1) infection pathway enrichment. Functional annotations highlight commonalities in protein roles. (**B**) PPI network of the top 10 hub genes within the term, identified using cytoHubba based on Maximal Clique Centrality (MCC) analysis. Node colors indicate MCC scores, from high (red) to low (yellow). (**C**) Bar plots of the top 10 hub genes by MCC score. Asterisks indicate genes annotated under IFN signaling, as shown in Figure 7A. Bar colors represent the predicted regulation of each gene in pregnancy based on differential miRNA expression: blue for downregulated, red for upregulated, and green for mixed regulation (both up- and downregulated). (**D**) miRNA–hub gene target networks show 25 nodes and 17 edges. Triangles represent miRNAs and rectangles represent hub genes. Node colors reflect inferred regulation in pregnancy: blue (downregulated), red (upregulated), and green (mixed).

**Figure 8:**
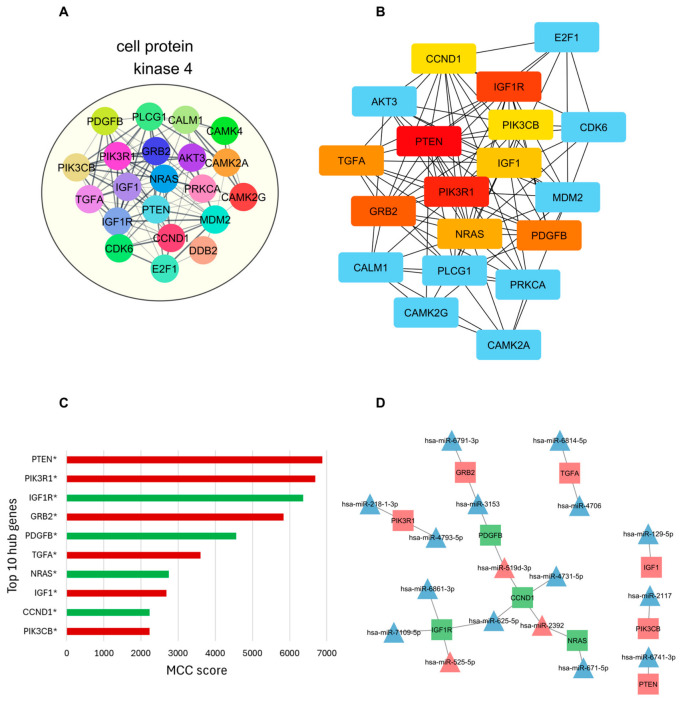
PPI and hub gene networks of HIV interactome genes within the glioma KEGG pathway, including predicted miRNA–target interactions. (**A**) Protein–protein interaction (PPI) networks display HIV interactome genes contributing to the glioma pathway enrichment. Functional annotations highlight commonalities in protein roles. (**B**) PPI network of the top 10 hub genes within the term, identified using cytoHubba based on Maximal Clique Centrality (MCC) analysis. Node colors indicate MCC scores from high (red) to low (yellow). (**C**) Bar plots of the top 10 hub genes by MCC score. Asterisks indicate genes annotated under cell protein kinase, as shown in Figure 8A. Bar colors represent the predicted regulation of each gene in pregnancy based on differential miRNA expression: blue for downregulated, red for upregulated, and green for mixed regulation (both up- and downregulated). (**D**) miRNA–hub gene target networks show 27 nodes and 21 edges. Triangles represent miRNAs and rectangles represent hub genes. Node colors reflect inferred regulation in pregnancy: blue (downregulated), red (upregulated), and green (mixed).

**Figure 9:**
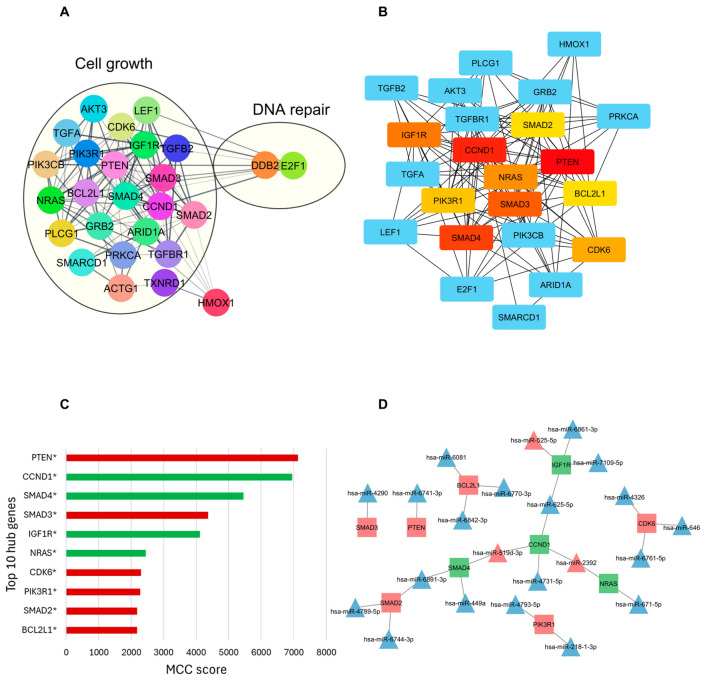
PPI and hub gene networks of HIV interactome genes within the Hepatocellular Carcinoma (HCC) KEGG pathway, including predicted miRNA–target interactions. (**A**) Protein–protein interaction (PPI) networks display HIV interactome genes contributing to the HCC pathway enrichment. Functional annotations highlight commonalities in protein roles. (**B**) PPI network of the top 10 hub genes within the term, identified using cytoHubba based on Maximal Clique Centrality (MCC) analysis. Node colors indicate MCC scores from high (red) to low (yellow). (**C**) Bar plots of the top 10 hub genes by MCC score. Asterisks indicate genes annotated under cell growth, as shown in Figure 9A. Bar colors represent the predicted regulation of each gene in pregnancy based on differential miRNA expression: blue for downregulated, red for upregulated, and green for mixed regulation (both up- and downregulated). (**D**) miRNA–hub gene target networks show 32 nodes and 26 edges. Triangles represent miRNAs and rectangles represent hub genes. Node colors reflect inferred regulation in pregnancy: blue (downregulated), red (upregulated), and green (mixed).

PPI networks built for genes targeted by pregnancy-driven miRNA overlapping with the HIV interactome contributed to six enriched and functionally mapped biological pathways. The “adaptive immune response” PPI network (Figure 4A) included 43 nodes and 80 edges mapped into two functional groups (*cellular receptors* and *DNA processes*), while “Cellular Senescence” comprised 35 nodes and 180 edges assigned to five functional groups (*cell cycle, calcium-activated phosphatase*, *ion channel*, *MAPK signaling and plasma membrane proteins*) (Figure 5A). “Hippo Signaling Pathway” included 23 nodes and 69 edges distributed into three functional groups (*TGF-β signaling*, *cellular housekeeping and protein phosphatases*) (Figure 6A). The “HSV-1 Infection” PPI network (Figure 7A) included 38 nodes and 77 edges mapped into five functional groups (*IFN signaling*, *antigen presentation and complement pathway, cellular processes*, *PI3K/AKT signaling pathway* and *serine/arginine-rich (SR) protein family*). The cancer-related pathways, “Glioma” and “HCC” included 21 nodes and 102 edges associated with *cell protein kinase* (Figure 8A), and 27 nodes and 131 edges linked to *Cell Growth and DNA repair, respectively* (Figure 9A). In summary, network mapping revealed essential functional groups that were likely altered during pregnancy.

Hub genes are highly connected within a PPI network. They are prime candidates for drug targeting, as their central roles within the network suggest they are likely essential for disease initiation and progression. To further explore this, we identified the top 10 hub genes that support and maintain the structural integrity of all PPI networks (Figure 4B, Figure 5B, Figure 6B, Figure 7B, Figure 8B and Figure 9B), presenting them according to their MCC scores and predicted direction of regulation, which is either upregulated, downregulated, or bidirectional (Figure 4C, Figure 5C, Figure 6C, Figure 7C, Figure 8C and Figure 9C). We identified pregnancy-driven miRNAs that regulate the hub genes and constructed their corresponding regulatory networks, highlighting the predicted miRNA–hub gene interactions within the following pathways: “adaptive immune response” (Figure 4D), “Cellular Senescence” (Figure 5D), “Hippo Signaling Pathway” (Figure 6D), “HSV1 Infection” (Figure 7D), “Glioma” (Figure 8D), and “HCC” (Figure 9D). Our data show a total of 47 unique hub genes across all the enriched pathways (Table 2) likely under post-transcriptional “one-to-many” and “many-to-one” regulation by 45 pregnancy-driven miRNAs, of which 40 are downregulated and five are upregulated. Moreover, C19MC-derived DE miRNAs target 12 top-ranked hub genes, representing 25.5% of all hub genes.

**Figure 10:**
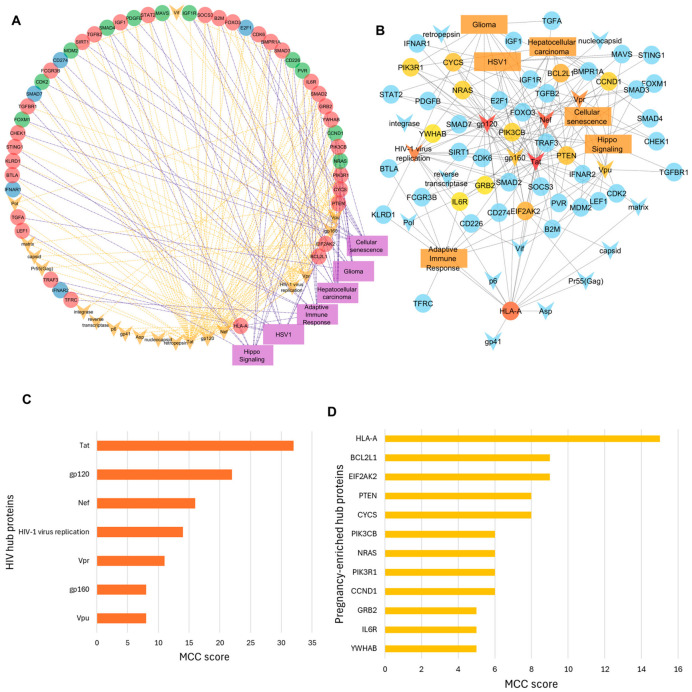
Circular network of hub genes, enriched pathways, and HIV-interacting modulators in pregnancy. (**A**) Ellipses represent hub genes with colors indicating their predicted regulation during pregnancy: downregulated (blue), upregulated (red), and mixed regulation (green). Enriched pathways are shown as purple rectangles, and HIV-interacting proteins are depicted as orange V-shapes. Orange dashed lines represent interactions between hub genes and HIV proteins, while purple dash-dot lines represent interactions between hub genes and enriched pathways. The network contains 72 nodes and 189 edges. (**B**) This subnetwork emphasizes highly connected HIV proteins (V-shapes), miRNA targets (circles), and enriched pathways (squares) extracted from panel A. The color gradient from intense orange to light yellow represents increasing Maximum Clique Centrality (MCC). All pathways in pregnancy showed an MCC = 10. (**C**) MCC score for HIV-1 proteins. (**D**) MCC for pregnancy-regulated host hub genes.

### 3.5. Host Hub Proteins Are Co-Targeted by Pregnancy-Driven miRNAs and Viral Proteins

Interactions between hub genes and HIV-1 proteins were sourced from the HIV-NCBI database [31]. These data were leveraged to construct a network incorporating the pathways associated with each hub gene (Figure 10A) and compiled into tables (Table 3, Table 4, Table 5, Table 6 and Table 7). We found that the hub genes were reported to interact with 19 HIV modulators, including an HIV biological process (HIV replication) and 18 HIV proteins (Capsid, gp120, gp160—precursor of gp120 and gp41, gp41, Integrase, Matrix, Nef, Nucleocapsid, p6, Pol, Pr55(gag)—(gag polyprotein precursor, Retropepsin—a protease, Reverse transcriptase, Tat, Vif, Vpr and Vpu). A subnetwork focuses on highly connected proteins (Figure 10B). Among these HIV-1 proteins, Tat and gp120 showed the highest connectivity within the network, followed by Nef, HIV-1 virus replication, Vpr, gp160, and Vpu (Figure 10C). HLA-A stood out as the most highly connected host protein, with BCL2L1 ranking second, followed by EIF2AK2, PTEN, and CYCS, completing the top five host proteins (Figure 10D).

## 4. Discussion

Our study leverages a robust cohort of women at higher risk of acquiring HIV throughout their reproductive cycles, providing a unique opportunity to investigate systemic molecular changes during pregnancy. Only a small number of prior longitudinal studies have reported peripheral blood miRNAs in pregnancy and primarily focus on preterm birth [36] and maternal blood pressure as underlying risk of adverse birth outcomes [37] and pre-eclampsia [38]. Our work specifically examines cell-free serum miRNAs and provides new insights into molecular mechanisms, potentially shaping HIV susceptibility and pathogenesis. By following women before and after pregnancy, we capture miRNAs that are likely critical during this period, revealing their systemic roles in pregnancy.

One of the several physiological adaptations that occur during pregnancy involves the suppression of immune functions, irrespective of the woman’s HIV status. These changes include early pregnancy events such as reduction in complement events and cell-mediated immunity [39]. In this sense, scientists and clinicians have yet to reach a consensus on whether pregnancy itself constitutes a risk factor for HIV acquisition [17,40,41,42]. However, a recent study analyzing data from two large clinical trials concluded that the HIV risk increases during pregnancy and continues through the first six months postpartum, with the highest risk occurring in the later stages of pregnancy [1]. While association studies have laid the groundwork in exploring the link between pregnancy and HIV risk, too little is known about this topic, and limited empirical molecular evidence has been proposed.

MiRNAs are well-known immune modulators that regulate gene expression and, consequently, contribute to immune-related processes [43]. In this study, we determined changes in serum miRNAs across pre-pregnancy, pregnancy, and breastfeeding reproductive states, providing evidence that molecular mechanisms governed by systemic miRNAs also undergo physiological adaptation during those transitions. We tested the hypothesis that those individual miRNAs regulate pivotal genes and pathways related to the HIV interaction, influencing susceptibility and progression during pregnancy.

It has been proposed that in pregnancy some miRNAs are expressed mainly from the placenta and trophoblast cells, in a coordinated manner from specific chromosomal clusters rather than individually [44,45]. The human placenta expresses two key imprinted microRNA clusters: the paternally expressed chromosome 19 microRNA cluster (C19MC) and the maternally expressed chromosome 14 cluster (C14MC). C19MC is highly placenta-specific, enriched in trophoblast-derived vesicles such as exosomes, and its members are detectable in maternal serum [46,47]. C14MC is known as the largest miRNA cluster and is primarily maternally expressed, showing dynamic regulation across gestation [44]. We identified 133 HIV interactome genes regulated by the DE placental cluster miRNAs circulating in the maternal serum, constituting a large proportion (~25%) of the hub genes with high connectivity to HIV proteins, all targeted by upregulated maternal-imprinted C19MC.

C19MC miRNAs have been shown to modulate maternal immune responses, promoting tolerance at the maternal–fetal interface [48]. In vitro studies have shown that while C19CM is not modulated by exposure to certain viruses, such as Vesicular Stomatitis Virus (VSV) and Cytomegalovirus (CMV) [49], it can be protective against ZIKV infection [50], vaccinia virus, VSV, HSV-1, but not CMV [51]. Whether and which specific miRNA members of C14MC and C19MC affect maternal HIV susceptibility and vertical transmission remain to be investigated.

Here, we reported the enrichment of six pathways in pregnancy. Among those, the Hippo Signaling pathway stands out as physiologically relevant during pregnancy due to its proteins’ role in early cell fate decisions between placental and embryonic lineages, as well as in trophoblast invasion [52,53], supporting the validity and biological relevance of our analysis. The Hippo Signaling pathway is functionally linked to the Cellular senescence pathway [54], suggesting coordinated regulation. Cellular senescence is also physiologically linked to cancer-associated pathways, such as HCC and glioma, as dysregulated cell cycle control is a hallmark of tumorigenesis [55,56]. The additional two pathways reflect over-representation of the adaptive immune response and viral infection processes, particularly those related to HSV-1. Curiously, a parallel has been proposed between maternal immune tolerance to the fetus during pregnancy and immune evasion mechanisms in cancer [57,58]. The idea is that the same immune system adaptations that help a fetus grow safely might also help cancer cells avoid being attacked. This might explain the cancer-related pathways found in this study and their linked genes. For instance, PDL-1—a known cancer suppressor, predicted to be downregulated in our dataset—is one of the top hub genes found in the “adaptive immune response” category. Blood levels of PDL-1 were similar in pregnant women without cancer and non-pregnant cancer patients when compared to healthy non-pregnant women [58]. Genes overlapping the HIV interactome are present across all enriched pathways. Therefore, our data highlights a gene network targeted by miRNAs DE in pregnancy that also shares similarities with cancer pathways and potentially interacts with HIV-1.

The pregnancy-enriched pathway analysis revealed protein–protein interaction networks converging on 47 hub genes shared with the HIV interactome. The miRNA–hub gene networks revealed “one-to-many” and “many-to-one” relationships, shedding light on a complex regulatory landscape in which individual miRNAs can influence multiple hub genes, while key hub genes may be co-regulated by several miRNAs. This reflects coordinated regulatory control over critical biological processes.

The hub genes identified in the “adaptive immune response” term converge on immune regulation with implications for tumor immunity, infection, and tolerance. They include immune checkpoints (PD-L1/CD274, BTLA, and PVR/CD155, the latter interacting with the co-stimulatory receptor CD226 [59]), a major histocompatibility complex (MHC) class I antigen processing and presentation machinery (HLA-A, B2M, TFRC), and additional inflammatory mediators such as IL6R and FCGR3B. Complementing these findings, hub genes linked to the “HSV1 infection” pathway fall into three categories: components of the type I interferon response (EIF2AK2, MAVS, TRAF3, STING1, STAT2, IFNAR1/2), negative feedback regulators (SOCS3), and apoptotic mediators (BCL2L1, CYCS), which together influence whether T cells, NK cells, and other immune populations undergo survival or programmed cell death, underscoring the integration of antiviral sensing with cytokine regulation and immune cell fate decisions.

Hub genes in the cellular senescence and Hippo Signaling pathways highlight coordinated regulation of cell fate. Senescence-associated genes balance cell cycle progression (CCND1, also in the Hippo Signaling pathway, CDK2/6, E2F1, FOXM1, CHEK1) with growth arrest and stress responses (PTEN, FOXO3, SIRT1, MDM2), while Hippo pathway genes are largely composed of TGF-β signaling components (SMAD2/3/4/7, TGFBR1, TGFB2, BMPR1A). Downstream effectors (LEF1 and YWHAB) integrate transcriptional programs controlling proliferation, differentiation, and tissue homeostasis. Together, they seem to reflect an interconnected network orchestrating proliferation versus arrest and survival decisions. The remaining three hub genes in both Glioma and HCC pathways act in growth factor signaling, likely with IGF1R upstream of NRAS and PIK3R1 driving proliferation and survival. Together, these hub genes may be key determinants of HIV risk during pregnancy and highlight three major themes—immune regulation, cell cycle control, and growth/homeostasis—targeted or dysregulated by HIV-1 as part of its immune evasion strategy.

The global miRNA transcriptome during pregnancy identified specific interactions between hub gene targets, host proteins, and HIV proteins in the HIV interactome. Among the host proteins, HLA-A, an MHC class I transmembrane protein, showed the highest number of interactions, while Tat emerged as the central HIV modulator, alongside gp120 and Nef. HLA-A, predicted to be upregulated, belongs to a gene complex that accounts for much of the observed variation in immune responses. It is highly polymorphic, and genetic variations have been demonstrated to be relevant in the vertical transmission of HIV [60,61]. A large study of ~9800 HIV-infected individuals found that elevated HLA-A expression was associated with significantly higher viral load and lower CD4^+^ T-cell counts, indicating poorer virologic control [62]. HIV Nef exhibits strong interaction with HLA-A and is known to downregulate its expression [63].

BCL2L1 (also known as BCL-2) was identified as the second most highly connected host protein within the HIV hub-gene network. HIV-infected cells are enriched in BCL-2, an anti-apoptotic factor that enhances their survival [64]. By sustaining the reservoir and reinforcing latency, BCL2L1 contributes to the long-term persistence of infection [65]. Pharmacologic inhibition of BCL-2 with venetoclax has been shown to promote the clearance of actively replicating HIV-infected cells in both in vitro and ex vivo models [66], and this approach is currently being evaluated in clinical trials (NCT05668026). In this context, miRNAs that regulate these pathways may offer additional opportunities for therapeutic intervention.

EIF2AK2, which is predicted to be upregulated, shows increased expression at late gestation in both circulating macrophages and placental tissues [67]. Activation of EIF2AK2 (PKR) by viral or RNA signals induces phosphorylation of eIF2α, a central antiviral pathway that normally limits protein synthesis. However, HIV can exploit this mechanism to enhance splicing of its rev/tat mRNA and promote productive viral gene expression [68]. In addition, the HIV Tat protein can compete with eIF2α, increasing transcription from the HIV-1 long terminal repeat (LTR), a regulatory DNA sequence [69]. EIF2AK2 also interacts with mitochondrial antiviral signaling proteins on the outer mitochondrial membrane, linking viral RNA sensing to mitochondrial-mediated immune responses. Notably, PKR has been implicated in placental immune regulation, suggesting a role in maintaining pregnancy homeostasis and responding to cellular stress. PTEN, also predicted to be upregulated in our dataset, appears to have a dual role in HIV infection. It can act as a barrier to viral replication by silencing unintegrated HIV-1 DNA [70], yet HIV integration into the PTEN gene may disrupt its function [71], potentially affecting immune responses. Investigating PTEN’s role during HIV exposure in pregnancy could provide valuable insights into maternal immune regulation and inform potential therapeutic strategies.

CYCS, an apoptotic protein predicted to be upregulated in our dataset, encodes cytochrome c, a mitochondrial factor central to placental function and pregnancy outcomes [72,73]. Mitochondrial dysfunction, marked by impaired cytochrome c oxidase activity and excessive cytochrome c-mediated apoptosis, has been linked to pre-eclampsia and related complications [74]. Elevated circulating cytochrome c has also been observed in HIV patients with antiretroviral therapy-related toxicity [75]. Cytochrome c is also central to HIV-1-induced apoptosis, in which viral proteins such as Env and Vpr directly or indirectly promote mitochondrial membrane permeabilization [76]. Thus, cytochrome c is likely a convergence point between pregnancy disorders and HIV pathogenesis.

Our results reveal a complex interplay in which pregnancy-associated miRNAs and HIV-1 proteins converge on shared hub genes, suggesting two-way co-targeting. This convergence points to a molecular tug-of-war between host regulatory mechanisms and viral interference during pregnancy. This overlap may reveal potential points of susceptibility in pregnant women, where physiological adaptations to pregnancy intersect with viral strategies to hijack host pathways. A number of experimental studies have provided evidence for direct interaction of circulating cell-free RNAs with HIV–host cells providing the basis of direct enhancement of HIV infection [77,78]. Future studies should validate key pregnancy-associated miRNA interactions with HIV in relevant models and explore their timing throughout gestation. Integrating additional non-coding RNAs and transcriptomic data may uncover broader regulatory networks, opening up new possibilities in informed targeted HIV prevention strategies tailored to reproductive stages.

## 5. Conclusions

Our longitudinal study reveals that systemic miRNAs undergo regulation during gestation, likely supporting maternal–fetal immune tolerance. We identified 47 hub genes targeted by these pregnancy-driven miRNAs (with 25% of those expressed by placental cluster C19MC) that overlap with the HIV interactome. Those genes include immune checkpoints (PD-L1, BTLA), apoptotic mediators (BCL2L1, CYCS), and antiviral signaling components (EIF2AK2, PTEN). Pregnancy-regulated miRNA converged on pathways of immune regulation, cell cycle control, and viral sensing, highlighting mechanisms by which HIV-1 may exploit pregnancy-associated molecular adaptations, or alternatively could be counteracted by host protective responses. Some of the differentially regulated direction of miRNAs mechanistically supported an increased risk of HIV-1 during pregnancy by targeting genes promoting host cell permissiveness (e.g., ↑ CD16) or viral replication (e.g., ↓ E2F1, ↑ FOXO3, ↑ CHEK-1). Other effects were bidirectional, holding wider potential to protect depending on the environmental context and exposures that have not been studied here. Future work should validate these interactions, establish the predictive value of miRNA signature for HIV-1 acquisition in longitudinal cohorts, and examine gestational timing to inform stage-specific HIV prevention or treatment strategies.

## Figures and Tables

**Table 1:** Participant characteristics and clinical variables.

Participant Demographics	Uganda	Zimbabwe	Total (N)
Women: n	40	48	88
Age at first visit (years)			
Mean ± SD	23.10 ± 3.49	24.02 ± 4.25	NA
Samples: n (%)			
Pre-pregnant	18 (34.6%)	34 (65.4%)	52
Pregnant	39 (44.3%)	49 (55.7%)	88
Breastfeeding	15 (44.1%)	19 (55.9%)	34
Total	72	102	174
Women with single/unpaired sampling: n			
Pre-pregnant only	1	0	1
Pregnant only	11	0	11
Breastfeeding only	0	0	0
Total	12	0	12
Women with longitudinal samples ^1^: n			
Pregnant and Pre-pregnant	13	30	43
Pregnant and Breastfeeding	11	15	26
Pre-Pregnant and Pregnant and Breastfeeding	4	4	8
Total	28	49	77
STI diagnoses at sample collection: n (%)			
HIV seroconversion ^2^	0 (0%)	5 (100%)	5
Chlamydiasis			
Positive	1 (1.4%)	2 (2%)	3
Negative	71 (98.6%)	100 (98%)	171
Gonorrhea			
Positive	3 (4.2%)	1 (1%)	4
Negative	69 (98.5%)	101 (99%)	170
Genital herpes			
Positive	50 (69.4)	70 (68.6%)	120
Negative	22 (30.6)	32 (31.4%)	54
Trichomoniasis			
Positive	1 (1.4%)	2 (2%)	3
Negative	70 (98.6%)	100 (98%)	170
Missing	1	0	
Other vaginal infections at sample collection: n (%)			
Candidiasis			
Positive	7 (9.7%)	16 (15.7%)	23
Negative	65 (90.3%)	83 (81.4%)	148
Missing	0	3 (2.9%)	3
Bacterial vaginosis (Nugent score)			
BV (7–10)	18 (25%)	28 (27.5%)	46
Intermediate (4–6)	10 (13.9%)	16 (15.7%)	26
Normal (0–3)	42 (58.33%)	51 (50%)	93
Missing	2 (2.8%)	7 (6.9%)	9

Abbreviations: STI = sexually transmitted infections, HIV = human immunodeficiency virus, HSV = herpes simplex virus, BV = bacterial vaginosis, NA = not applicable. ^1^ One woman from Zimbabwe cohort contributed during 2 pregnancies = 1 PP and P and BF (3 samples) and 1 PP and P (2 samples). ^2^ All women were HIV-negative at the preconception visit but five women from Zimbabwe cohort HIV-converted during pregnancy (n = 3) or breastfeeding (n = 2).

**Table 2:** Hub genes with top MCC scores featuring pregnancy-enriched pathways overlapping with the HIV interactome.

Sharing Status	Pathways	Hub Genes Symbol *
Unique to each biological pathway	Adaptive immune response	TFRC, **IL6R**, BTLA, B2M, CD226 **, PVR **, **HLA-A,** KLRD1, CD274 **, FCGR3B
	Cellular Senescence	CHEK1, FOXO3, MDM2 **, SIRT1, E2F1, FOXM1, CDK2
	Hippo Signaling	**YWHAB**, LEF1, BMPR1A, TGFB2, TGFBR1, SMAD7 **
	HSV1 Infection	STING1, **CYCS**, SOCS3, TRAF3, IFNAR2 **, IFNAR1 **, MAVS **, STAT2, **EIF2AK2**
	Glioma	**PIK3CB**, IGF1, TGFA, PDGFB **, **GRB2**
Shared by indicated pathways	Glioma and HCC	**NRAS**, IGF1R **, **PIK3R1**
	Hippo Signaling and HCC	SMAD2, SMAD3, SMAD4 **
	HSV1 Infection and HCC	**BCL2L1**
	Cellular Senescence and HCC	CDK6
	Cellular Senescence, Glioma and HCC	**PTEN**
	Cellular Senescence, Hippo Signaling, Glioma and HCC	CCND1 **

* Bolded hub gene symbols indicate those with strong interactions with HIV proteins, with Maximum Clique Centrality (MCC) score > 4.5, featured in Figure 10. ** Hub genes targeted by pregnancy upregulated miRNAs pertaining to Chromosome 19 miRNA Cluster (C19MC).

**Table 3:** Interactions between HIV-1 and the top 10 adaptive immune response hub genes ranked by Maximum Clique Centrality Score (MCC).

Hub Gene Rank by MCC	Hub Gene Symbol *	Hub Gene Name	HIV-1 Protein–Host Hub Gene Interactions as Reported in HIV-NCBI Database
1	↑ FCGR3B/CD16	Fc gamma receptor IIIb	▪ Tat-upregulated CD16 promotes HIV permissiveness of macrophages
2	↓ CD274/PDL1	CD274 molecule	▪ Envelope gp120 upregulates PDL1 in plasmacytoid dendritic cells as an invasion tactic ▪ Tat-upregulated PDL1 impedes T-cell proliferation
3	↑ KLRD1	Killer cell lectin-like receptor D1	▪ HIV-1 replication upregulates KLRD1 in mononuclear cells
3	↑ HLA-A	Major histocompatibility complex, class I, A	▪ Nef interacts with HLA-A in multiple ways, including degradation, binding, inhibition and downregulation, as a viral evasion tactic ▪ Vpu downregulates HLA-A in infected cells ▪ Tat is reported to down- and upregulate HLA-A ▪ Vpr upregulates HLA-A in macrophages ▪ gp120/gp41 upregulates HLA-A expression
4	↑↓ PVR/CD155	PVR cell adhesion molecule	▪ Nef andVpu downregulate PVR, escaping NK cell-mediated killing of HIV-infected cells ▪ Vpr upregulates PVR expression in T cells
4	↑↓ CD226	CD226 molecule	▪ Nef reduces CD226/PVR interaction leading to decreased NK cell-mediated lysis of HIV-infected cells. ▪ HIV-1 replication and Vif downregulate CD226 in T cells
5	↑ B2M	Beta-2-microglobulin	▪ Tat, Nef and Vpu downregulate B2M, a component of HLA
6	↑ BTLA	B and T lymphocyte associated	▪ Envelope gp120 downregulates BTLA expression in human B cells
7	↑ IL6R	Interleukin 6 receptor	▪ gp120, gp160 upregulate IL6R in human B cells ▪ Tat upregulates IL6R in human epithelial cells lineages
7	↑ TFRC	Transferrin receptor	Unknown §

* Hub gene regulation predicted as upregulation ↑, downregulation ↓ or bidirectional ↑↓ in accordance with DE miRNA in pregnancy; all hub genes are annotated under cellular receptor-related functions (Figure 4A). § interactions reported in Luo et al. [30]. NCBI: National Center for Biotechnology Information.

**Table 4:** Interactions between HIV-1 and cellular senescence hub genes ranked by Maximum Clique Centrality Score (MCC).

Hub Gene Rank by MCC	Hub Gene Symbol *	Hub Gene Name	HIV-1 Protein–Host Hub Gene Interactions as Reported in HIV-NCBI Database
1	↑↓ CCND1 ‡	Cyclin D1	▪ Nef upregulates Cyclin D1 inducing podocytes proliferation ▪ Tat’s context-dependent effects both up- and down-regulatie Cyclin D1 expression
2	↑↓ CDK2	Cyclin-dependent kinase 2	▪ Tat stimulates CDK2/Cyclin E activity enhancing viral replication ▪ CDK2 phosphorylates Tat; regulates HIV-1 transcription and host cell cycle progression
3	↑ PTEN §	Phosphatase and tensin homolog	▪ Tat up- or downregulates PTEN’s expression depending on cell type and context ▪ Nef downregulates PTEN promoting angiogenesis ▪ PTEN is required for HIV-1gp120-mediated neurotoxicity ▪ HIV matrix proteins activate PTEN and AKT pathways
4	↑ CDK6 †	Cyclin-dependent kinase 6	▪ Tat reduces CDK6 abundance in T cells ▪ HIV-1 infection downregulates CDK6 expression in T cells
5	↑↓ FOXM1	Forkhead box M1	▪ Vpr downregulates FOXM1 expression in astrocytes
6	↓ E2F1	E2F transcription factor 1	▪ E2F1 is required for neuronal cell death induced by HIV-1 gp120 through neuronal CXCR4 signaling ▪ E2F1 inhibits Tat-induced activation of the HIV-1 promoter
7	↑ SIRT1	Sirtuin 1	▪ Tat binds and inhibits SIRT1 promoting HIV-1 LTR transactivation
8	↑↓ MDM2	MDM2 protooncogene	▪ MDM2 ubiquitinates Tat, promoting HIV replication ▪ Vif binds MDM2 and inhibits its activity ▪ MDM2 degrades Vif, inhibiting HIV replication
9	↑ FOXO3	Forkhead box O3	▪ Tat increases nuclear FOXO3a in T cells ▪ Vpr inhibits insulin/Akt-induced FOXO3 translocation ▪ FOXO3 supports HIV replication
10	↑ CHEK1	Checkpoint kinase 1	▪ Vpr mediates chk-1 phosphorylation thus promoting viral replication

* Hub gene regulation predicted as upregulation ↑, downregulation ↓ or bidirectional ↑↓ in accordance with DE miRNA in pregnancy. All genes annotated under cell cycle-related functions (Figure 5A). Shared with other functional networks: ‡ with Hippo Signaling (Figure 6C), glioma (Figure 8C) and Hepatocellular Carcinoma (HCC) (Figure 9C), § with HCC, † with glioma and HCC. NCBI: National Center for Biotechnology Information.

**Table 5:** Interactions between HIV-1 and Hippo Signaling Pathway hub genes ranked by Maximum Clique Centrality Score (MCC).

Hub Gene Rank by MCC *	Hub Gene Symbol **	Hub Gene Name	HIV-1 Protein–Host Hub Gene Interactions as Reported in HIV-NCBI Database
1	↑↓ SMAD4 §	SMAD family member 4	▪ Tat downregulates SMAD4 phosphorylation; enhances SMAD4 binding to viral DNA ▪ SMAD4 inhibits Tat-induced transcription ▪ Tat downregulates SMAD4
2	↑ SMAD3 §	SMAD family member 3	▪ Tat’s interactions with SMAD3 are context-dependent, including altered phosphorylation, enhanced transcriptional complex binding, and reciprocal regulation of Tat-mediated transcription ▪ Vpr impairs NK cell function through cytokine activation of the SMAD3 signaling pathway ▪ SMAD3 can inhibit Tat transcription in some contexts
3	↓ SMAD7	SMAD family member 7	▪ gp120 upregulates SMAD7 expression in B cells and enhances apoptosis via p300
4	↑ SMAD2 §	SMAD family member 2	▪ gp120 induces SMAD2 phosphorylation leading to apoptosis ▪ Tat alters SMAD2 phosphorylation and enhances SMAD2-associated transcriptional complex binding. ▪ SMAD2 restricts HIV-1 replication in Langerhans cells
5	↑ TGFBR1	Transforming growth factor beta receptor 1	▪ Vpu promotes profibrogenic activation in LX2 cells through TGFβ signaling
6	↑ TGFB2	Transforming growth factor beta 2	▪ Vpu promotes TGF-β release ▪ Tat upregulates TGFB2 in epithelial cells and macrophages
8	↑ BMPR1A	Bone morphogenetic protein receptor type 1A	▪ Tat increases BMPR1A mRNA but reduces correlated protein
9	↑ LEF1	Lymphoid enhancer binding factor 1	▪ Tat suppresses LEF1
10	↑ YWHAB	Tyrosine 3monooxygenase/tryptophan 5monooxygenase activation protein beta	▪ gp120 upregulates YWHAB ▪ Vpr inhibits YWHAB interactions affecting G2 arrest and FOXO3a ▪ HIV downregulates YWHAB expression in T cells

* The gene ranked 7th is ↑↓ *CCND1*—not shown here because it is already shown in Table 4. ** Hub gene regulation predicted as upregulation ↑, downregulation ↓ or bidirectional ↑↓ in accordance with DE miRNA in pregnancy; all genes annotated in TGFβ signaling-related functions (Figure 6A), except YWHAB, § are shared with the Hepatocellular Carcinoma Pathway. NCBI: National Center for Biotechnology Information.

**Table 6:** Interactions between HSV1 and hub genes ranked by Maximum Clique Centrality Score (MCC).

Hub Gene Rank by MCC	Hub Gene Symbol *	Hub Gene Name	HIV-1 Protein–Host Hub Gene Interactions as Reported in HIV-NCBI Database
1	↑ EIF2AK2	Eukaryotic translation initiation factor 2 alpha kinase 2	▪ Tat shows multiple context-dependent interactions with EIF2AK2, including activation, inhibition, binding, phosphorylation, and reciprocal regulation ▪ Interaction with Tat, Nef, gp120, gp160, Vpu, Vif, Pr55(Gag), and capsid
2	↑ STAT2	Signal transducer and activator of transcription 2	▪ Nef induces STAT2 phosphorylation ▪ gp120 activates STAT2 in primary human monocyte-derived dendritic cells
3	↓ IFNAR1	Interferon alpha and beta receptor subunit 1	▪ gp120 upregulates IFNAR1 in macrophages
4	↑↓ MAVS	Mitochondrial antiviral signaling protein	▪ Nef and Vpu block and degrade MAVS-induced antiviral signaling (IFN release) of T cells
5	↑ TRAF3	TNF receptor-associated factor 3	▪ Tat downregulates TRAF3 in microglial cells
6	↓ IFNAR2	Interferon alpha and beta receptor subunit 2	▪ Tat upregulates IFNAR2 in macrophages
7	↑ SOCS3	Suppressor of cytokine signaling 3	▪ gp120 recruits SOCS3 while Tat both upregulates and downregulates SOCS3
8	↑ BCL2L1 §	BCL2 like 1	▪ Nef, gp120, Vpu, Vpr, and nucleocapsid downregulate BCL2L1 ▪ Nef, Tat, and Vpr also upregulate or interact with BCL2L1
9	↑ STING1	Stimulator of interferon response cGAMP interactor 1	▪ Vpr antagonizes STING1-mediated innate immune recognition in STING-expressing TZM-bl cells
9	↑ CYCS	Cytochrome c, somatic	▪ Tat, Vpr, gp120, gp160, retropepsin, and Nef promote cytochrome c release ▪ Tat, retropepsin, and Nef interact with or upregulate CYCS. ▪ HIV-1 replication is inhibited by CYCS knockdown

* Hub gene regulation predicted as upregulation ↑, downregulation ↓ or bidirectional ↑↓ in accordance with DE miRNA in pregnancy, all genes annotated under interferon (IFN) signaling (Figure 7A), except SOCS3 and BCL2L1; § Shared with Hepatocellular Carcinoma Pathway. NCBI: National Center for Biotechnology Information.

**Table 7:** Interactions between HIV-1 and glioma hub genes ranked by Maximum Clique Centrality Score (MCC).

Hub Gene Rank by MCC *	Hub Gene Symbol **	Hub Gene Name	HIV-1 Protein–Host Hub Gene Interactions as Reported in HIV-NCBI Database
2	↑ PIK3R1 §	Phosphoinositide3kinase regulatory subunit 1	▪ Nef activates the PI3K/Akt/mTOR pathway ▪ gp120 activates PI3K and calcium mobilization ▪ Tat activates PI3K/Akt signaling ▪ Vpr stimulates PI3K/Akt/NFκB signaling in astrocytes
3	↑↓ IGF1R	Insulin-like growth factor 1 receptor	▪ HIV-1 gp120 upregulates IGF1R expression, along with multiple other pro-survival and apoptotic pathway genes, in human neuronal cells
4	↑ GRB2	Growth factor receptor bound protein 2	▪ Tat interacts with GRB2. ▪ Tat promotes Ras activation via recruitment of GRB2, and upregulates GRB2 isoform Grb33, enhancing HIV1 LTR promoter activity. ▪ HIV-1 Pol and reverse transcriptase physically interact with GRB2 in human cell lines
5	↑↓ PDGFB	Platelet-derived growth factor subunit B	▪ PDGFB inhibits gp120 and Tat-induced neurotoxicity ▪ gp120 and Tat upregulate PDGFB expression in endothelial cells
6	↑ TGFA	Transforming growth factor alpha	▪ Tat upregulates transcription of the TGFA in an epidermal growth factor-dependent manner
7	↑↓ NRAS §	NRAS protooncogene, GTPase	▪ Nef and Tat activate NRAS signaling linked to cellular compartments and kinase cascades ▪ gp160 can both activate or inhibit NRAS ▪ NRAS supports HIV replication in T cells
8	↑ IGF1	Insulin-like growth factor 1	▪ IGF1 inhibits gp120 by competing with its binding to the CD4 receptor ▪ IGF1 inhibits Tat-mediated transactivation of the LTR promoter, ▪ Tat upregulates IGF1 and other anti-apoptotic genes in stressed Kaposi’s sarcoma cells.
9	↑ PIK3CB	Phosphatidylinositol4,5bisphosphate 3kinase catalytic subunit beta	▪ Tat and Vpr proteins manipulate the PI3K/Akt pathway to promote cell survival ▪ gp120/gp160 engages chemokine receptors (CCR5/CXCR4) ▪ Nef activates the PI3K/Akt/mTOR pathway

* The hub genes ranked 1st (PTEN) and 9^TH^ (CCND1) are not shown here because they are shown in Table 4. ** Hub gene regulation predicted as upregulation ↑ or bidirectional ↑↓ in accordance with DE miRNA in pregnancy; all genes annotated under cell protein kinase (Figure 8A), § are shared with Hepatocellular Carcinoma Pathway. NCBI: National Center for Biotechnology Information.

## Data Availability

All data are provided in Supplemental Tables.

## Supplementary Materials

The following supporting information can be downloaded at: https://www.mdpi.com/article/10.3390/v18070753/s1, Table S1: List of miRNAs gene symbols used in the HTG assay; Table S2: List of genes pertaining to the HIV interactome dataset; Table S3: Serum miRNAs differentially expressed in Pregnancy versus Pre-pregnancy at FDR < 0.1; Table S4: Differentially expressed serum miRNAs in pregnancy (FDR < 0.1) identified consistently across P vs. PP and P vs. BF comparisons; Table S5: Validated targets of serum miRNAs upregulated in Pregnancy; Table S6: Venn diagram analysis of overlap between the HIV interactome and proteins predicted to be downregulated or upregulated in pregnancy; Table S7: Functional enrichment analysis of pregnancy-altered genes based on KEGG and Gene Ontology terms; Table S8: Genes in pregnancy-enriched pathways targeted by DE-miRNAs and shared with the HIV interactome; Table S9: Proportion of genes overlapping the HIV interactome per pregnancy-enriched pathway. Supplemental Figure S1: Principal Component Analysis (PCA) for comparisons of Pregnant vs. Pre-pregnant Breastfeeding vs. Pregnant groups, including batch effect.

## Abbreviations

The following abbreviations are used in this manuscript:

BF	Breastfeeding
BV	Bacterial vaginosis
CMV	Cytomegalovirus
CPM	Counts per million
DE	Differentially expressed
FC	Fold change
FDR	False Discovery Rate
GO	Gene Ontology
GOIS	Gene Ontology of Immune System
HCC	Hepatocellular carcinoma
HIV	Human immunodeficiency virus
HSV	Herpes simplex virus
KEGG	Kyoto Encyclopedia of Genes and Genomes
MC	miRNA cluster
MCC	Maximum Clique Centrality
MEA	Modular Enrichment Analysis
miRWT	miRNA whole transcriptome
NGS	Next-generation sequencing
P	Pregnant
PCA	Principal component analysis
PP	Pre-pregnant
PPI	Protein–protein interaction
STIs	Sexually transmitted infections
VSV	Vesicular stomatitis virus
