# Supplementary material for: MicroRNAs Regulated by Pregnancy Target Antiviral and Cancer Immunity Overlapping with the HIV Interactome
Source: Viruses. 2026 Jul 7;18(7):753. doi: 10.3390/v18070753 (PMC13431474; doi:10.3390/v18070753)
Supplement: Supplementary file 1 [file viruses-18-00753-s001.zip › Supplemental Figure S1.pdf]

A

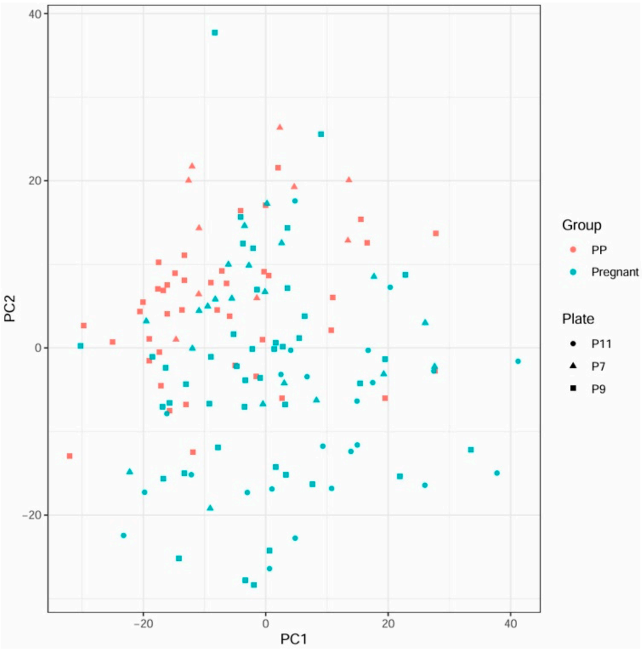

B

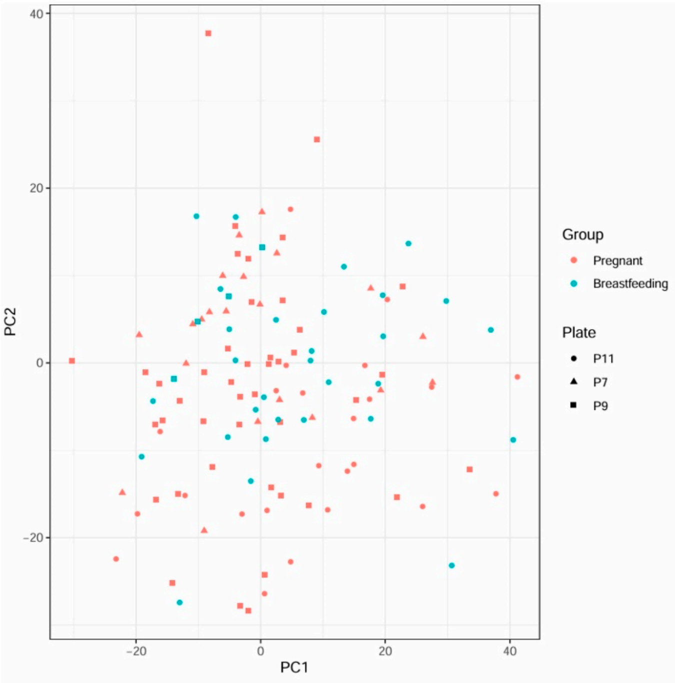

Supplemental Figure S1: Serum miRNA expression by female reproductive status. Principal Component Analysis (PCA) was performed for (**A**) Pregnant (P) vs. Pre-pregnant (PP) and (**B**) Breastfeeding (BF) vs. Pregnant groups, including batch effect (Plate).
